# Clinical Relevance of PD-L1 Expression and CD8+ T Cells’ Infiltration in Patients With Lung Invasive Mucinous Adenocarcinoma

**DOI:** 10.3389/fonc.2021.683432

**Published:** 2021-06-24

**Authors:** Xiaoling Xu, Na Li, Ding Wang, Wei Chen, Yun Fan

**Affiliations:** ^1^ Key Laboratory of Head & Neck Cancer Translational Research of Zhejiang Province, Cancer Hospital of the University of Chinese Academy of Sciences (Zhejiang Cancer Hospital), Hangzhou, China; ^2^ Institute of Cancer and Basic Medicine (IBMC), Chinese Academy of Sciences, Hangzhou, China; ^3^ Department of Medical Oncology, The Second Clinical Medical College of Zhejiang Chinese Medical University, Hangzhou, China

**Keywords:** invasive mucinous adenocarcinoma, lung cancer, PD-L1 expression, CD8+ T cells, tumor microenvironment, genetic characteristics, treatment

## Abstract

**Background:**

Invasive mucinous adenocarcinoma (IMA) of the lung is a rare and distinct subtype of adenocarcinoma. At present, people have no idea whether IMA patients can benefit from immunotherapy and target therapy; thus there is an urgent need to clarify the immune microenvironment and genetic characteristics of this cohort.

**Methods:**

A total of 31 IMA patients matched with 27 non-mucinous adenocarcinoma (non-IMA) patients were enrolled in this study, and clinical data was collected. The expression of PD-L1, CD8+ tumor-infiltrating lymphocytes (TILs) and ALK was determined by immunohistochemistry. Polymerase Chain Reaction was used to determine the mutations of EGFR. The Chi-square test, Kaplan–Meier method and Cox proportional hazard regression model were used to explore the correlations between these clinicopathological variables, survival and identify risk factors.

**Results:**

Of the patients with IMA 9.7% (3/31) revealed positive PD-L1 expression and 35.5% (11/31) showed CD8+ TIL infiltration, which were markedly lower than that of non-IMA group [PD-L1: 48.1% (13/27); CD8: 81.5% (22/27)]. Moreover, five (16.1%) patients in IMA group and 10 (37.0%) patients in non-IMA group had EGFR mutations, and nine (29.0%) patients in IMA group and zero (0.0%) patient in non-IMA group had ALK rearrangements. Additionally, we observed that IMA patients with CD8+ TIL infiltration had a worse prognosis than CD8-negative group (P = 0.024). Multivariate analyses showed that CD8 was an independent prognostic factor for patient’s survival (HR = 5.60, 95% CI: 1.35–23.22, P = 0.017).

**Conclusion:**

Patients with IMA have down-regulated expression of PD-L1 and less CD8+ TIL infiltration in tumor microenvironment. Besides, a lower frequency of EGFR mutations was detected in patients with IMA than non-IMA patients while a higher rate of ALK rearrangements was found. Our results provide important reference for therapy of lung IMA.

## Introduction

Lung cancer remains the leading cause of cancer-related mortality worldwide which can be pathologically classified into two major subtypes, non-small cell lung cancer (NSCLC) and small cell lung cancer (SCLC) (1). Lung adenocarcinoma (ADC) is the most frequent type of NSCLC. According to the new classification proposed by the European Respiratory Society (ERS), American Thoracic Society (ATS) and International Association for the Study of Lung Cancer (IASLC) in 2011, invasive mucinous adenocarcinoma of the lung (IMA) is a rare and unique lung ADC subtype, about 2–5% (2). IMA is histologically characterized by goblet and/or columnar cells with basal nuclei and abundant cytoplasmic mucin (3). As for prognosis, IMA is related with poor prognosis mainly owing to airway propagation mode (4). In addition, pathological parameters, including tumor cell spread size, invasive size, and mucin spread size, were also adverse prognostic factors for IMA (5). As a subtype of lung ADC, the same therapeutic regimen as for other ADCs was usually applied for treating patients with IMA. However, neither platinum-based chemotherapy nor targeted therapy has been demonstrated to be obviously effective against IMA (6, 7). Thus, new therapeutic approach for IMA is necessary.

IMA has a special gene expression profiling. Recent research studies have proved that KRAS mutation was the most frequent oncogenic driver mutations in IMA (63–90%) followed by NRG1 fusions (7–27%) (8–11). Compared with non-mucinous adenocarcinoma (non-IMA), IMA has a lower rate of EGFR mutations (only 0–5%) and a higher rate of ALK rearrangements (2.2%) and ERBB2 mutations (1.2%) (6, 12, 13). In addition, rare gene mutations, such as HER2, BRAF, and PI3KA mutations, and rare gene fusions, such as TRIM4-BRAF, VAMP2-NRG1, and CD74-NRG1fusions, were observed in IMA patients with KRAS-negative (8). However, owing to the rarity of treatable mutations, IMA patients are usually ineligible for target therapy (6).

Recently, immune checkpoint inhibitors (ICIs) have greatly changed the treatment landscape of patients with non-small cell lung cancer (14). However, due to the rarity of IMA, the studies of immune-checkpoint expression in patients with IMA have been limited, and no specific immune checkpoint therapy has been established for IMA yet (15). Nakagomi et al. (7) found that PD-L1 expression tended to be lower in the IMA group (6.1%) compared to the conventional ADC group (59.7%). Another study (16) detect PD-L1 expression in NSCLC including various adenocarcinoma subtypes. Out of the 90 samples, only four were IMA and none of them expressed PD-L1. In general, the PD-L1 expression in IMA patients was rather low and, in fact, whether IMA patients can benefit from ICIs still needs further investigation. Moreover, B7-H3 expressed highly in IMA group (42.4%), which maybe a potential and promising immunotherapeutic target.

Based on the available literature, although IMA is a variant of lung ADC, it has specific genetic profiles and immune-checkpoint status, which means that innovative therapies are needed for this subgroup. Unfortunately, there are only a few studies relevant. In our study, by reviewing the clinicopathological features, genetic mutations, tumor microenvironment (TME), and survival of 31 IMA patients, we aimed to clarify PD-L1 expression and tumor-infiltrating lymphocytes (TILs) in IMA, the correlation between these factors and patient’s survival, and the potential of targeted therapy and immunotherapy in IMA patients.

## Materials and Methods

### Study Population

A total of 9,260 patients with lung cancer were reviewed; only 148 patients with lung IMA were confirmed from January 2010 to December 2015 at Zhejiang Cancer Hospital. IMA patients who satisfied all of the following criteria were enrolled: (1) All collected tumor samples must be pathologically diagnosed as IMA; (2) All patients had complete clinical and follow-up information; (3) All patients did not receive any anti-tumor treatment; and (4) All patients signed informed consent. As a control group, NSCLC patients with non-IMA were also included. The clinical characteristics of the participants are listed in **Table 1**. The study was conducted in accordance with the ethical standards of the Ethics Committee of Zhejiang Cancer Hospital, and informed consent for the use of tumor specimens was obtained.

**Table 1:** Clinicopathological characteristics of the 58 patients with lung adenocarcinoma.

	All case (n = 58)	IMA (n = 31)	non-IMA (n = 27)	*P*-value
Sex				
Male	27 (46.6%)	15 (48.4%)	11 (40.7%)	0.408
Female	31 (53.4%)	16 (51.6%)	16 (59.3%)	
Age				
<65	47 (81.0%)	25 (80.6%)	22 (81.5%)	0.935
≥65	11 (19.0%)	6 (19.4%)	5 (18.5%)	
Smoking status				
Never	36 (62.1%)	19 (61.3%)	17 (63.0%)	0.896
Ever/current	22 (37.9%)	12 (38.7%)	10 (37.0%)	
Clinical stage				
I–III	45 (77.6%)	27 (87.1%)	18 (66.7%)	0.063
IV	13 (22.4)	4 (12.9%)	9 (33.3%)	
EGFR status				
Mutation	15 (25.9%)	5 (16.1%)	10 (37.0%)	0.070
Wild	43 (74.1%)	26 (83.9%)	17 (63.0%)	
ALK status				
Mutation	9 (15.5%)	9 (29.0%)	0 (0.0%)	0.002*
Wild	49 (82.8%)	22 (71.0%)	27 (100.0%)	
PD-L1 expression				
+ (≥1%)	16 (27.6%)	3 (9.7%)	13 (48.1%)	0.001*
− (<1%)	42 (72.4%)	28 (90.3%)	14 (51.9%)	
CD8 expression				
+ (≥10%)	33 (56.9%)	11 (35.5%)	22 (81.5%)	<0.001*
− (<10%)	25 (43.1%)	20 (64.5%)	5 (18.5%)	

*P-value < 0.05 in Chi-square test. In bold: P < 0.05.

### Immunohistochemistry

Immunohistochemistry (IHC) assay was performed in tumor samples comprising 41 surgical samples and 17 biopsies. The 4-µm tissue sections were cut from formalin-fixed paraffin-embedded (FFPE) tumor tissues. After deparaffinization and rehydration, slides were stained in an automated system (Leica Biosystems, Wetzlar, Germany). Antibodies used were rabbit anti-PD-L1 (1:100, clone SP142, cat# ZA-0629, Beijing zhongshan Jinqiao Biotechnology Co., Ltd, Beijing, China), mouse anti-CD8 (1:100, clone ES05, cat# IR079, Dako, Agilent Technologies, Santa Clara, CA, USA), and rabbit anti-ALK (1:100, clone D5F3, cat# 3633S, Cell Signaling Technology, MA, USA).

PD-L1 expression was evaluated by the tumor proportion score (TPS), defined as the percentage of tumor cells observed as partial or complete membrane staining. The cut-off for PD-L1 positive expression was set at ≥1%. The positivity of CD8+ T cells of all nucleated cells in the intercellular substance was defined as ≥10%. PD-L1+/CD8+ expression was defined as both positive expression of PD-L1 and CD8+ T cell.

ALK expression was determined using binary interpretation. We defined ALK positivity as any percentage of presence of strong granular cytoplasmic staining in the tumor cells; otherwise, the absence of strong cytoplasmic staining was deemed ALK negative. IHC assay was performed according to the manufacturer’s protocols, and two pathologists independently scored staining with blind assessments.

### EGFR Mutation Analyses

DNA was extracted from the FFPE tumor tissues using Amoydx FPE DNA Kit (Amoy Diagnostics, Xiamen, China) according to protocols. Polymerase chain reaction (PCR) was performed at the Mx3000PTM real-time PCR system (Strata gene, La Jolla, USA). EGFR mutations were detected by EGFR 29 Mutations Detection Kit (Amoy Diagnostics, Xiamen, China). We used ΔCt method to quantify the amplification. If ΔCt values were higher than 2.0, the patient was identified as “EGFR mutant”; otherwise, the patient was identified as “EGFR wild-type”.

### Statistical Analyses

To compare categorical characteristics, the Chi-square test was performed. Survival analyses were performed by plotting Kaplan–Meier curves with the log-rank test, and the hazard ratio (HR) was determined by multivariable Cox proportional hazard regression model. Variables included in this model were sex, age, smoking status, clinical stage, EGFR and ALK status, PD-L1, and CD8 expression. P value ≤0.05 was considered statistically significant. The data were statistically analyzed by using SPSS software, GraphPad Prism (version 5), and version 22.0 for Windows (Chicago, IL, USA).

## Results

### Patient Characteristics

**Figure 1:**
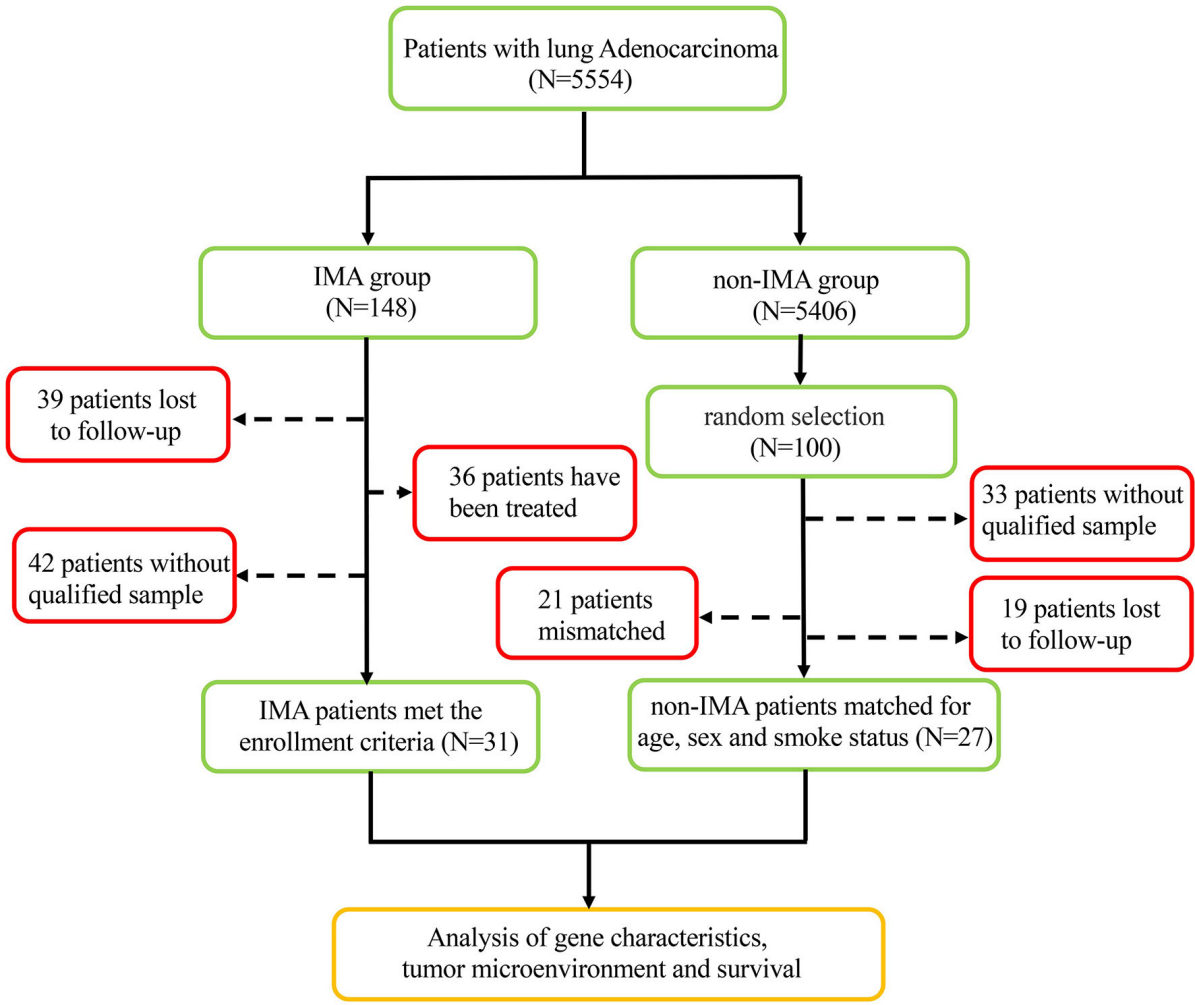
Flow chart of the protocol followed for patients’ enrollment.

From a screened population of 9,260 patients with lung cancer, a total of 31 IMA patients were identified; see flow chart in **Figure 1**. The non-IMA group consisting of 27 individuals was matched with the IMA group for age, sex, and smoking status. The median age of patients at diagnosis was 58 years (range: 29–85 years); 27 (46.6%) were men and 31 (53.4%) were women. Among them, 81.0% (47 patients) were <65 years, 37.9% (22 patients) were have a history of smoking, and 22.4% (13 patients) were diagnosed at stage IV. There were no significant differences between the IMA and non-IMA groups in terms of sex, age, smoking, and clinical stage. Five (16.1%) patients in IMA group, and 10 (37.0%) patients in non-IMA group had EGFR mutations, and nine (29.0%) patients in IMA group and zero (0.0%) patient in non-IMA group had ALK rearrangements. The ALK rearrangements rate were significantly higher in the IMA group (P = 0.002). Clinical and pathological characteristics of this cohort were presented in **Table 1**.

### Correlation With the Clinicopathology and Prognosis of PD-L1 Expression and CD8+ TIL Status

**Figure 2:**
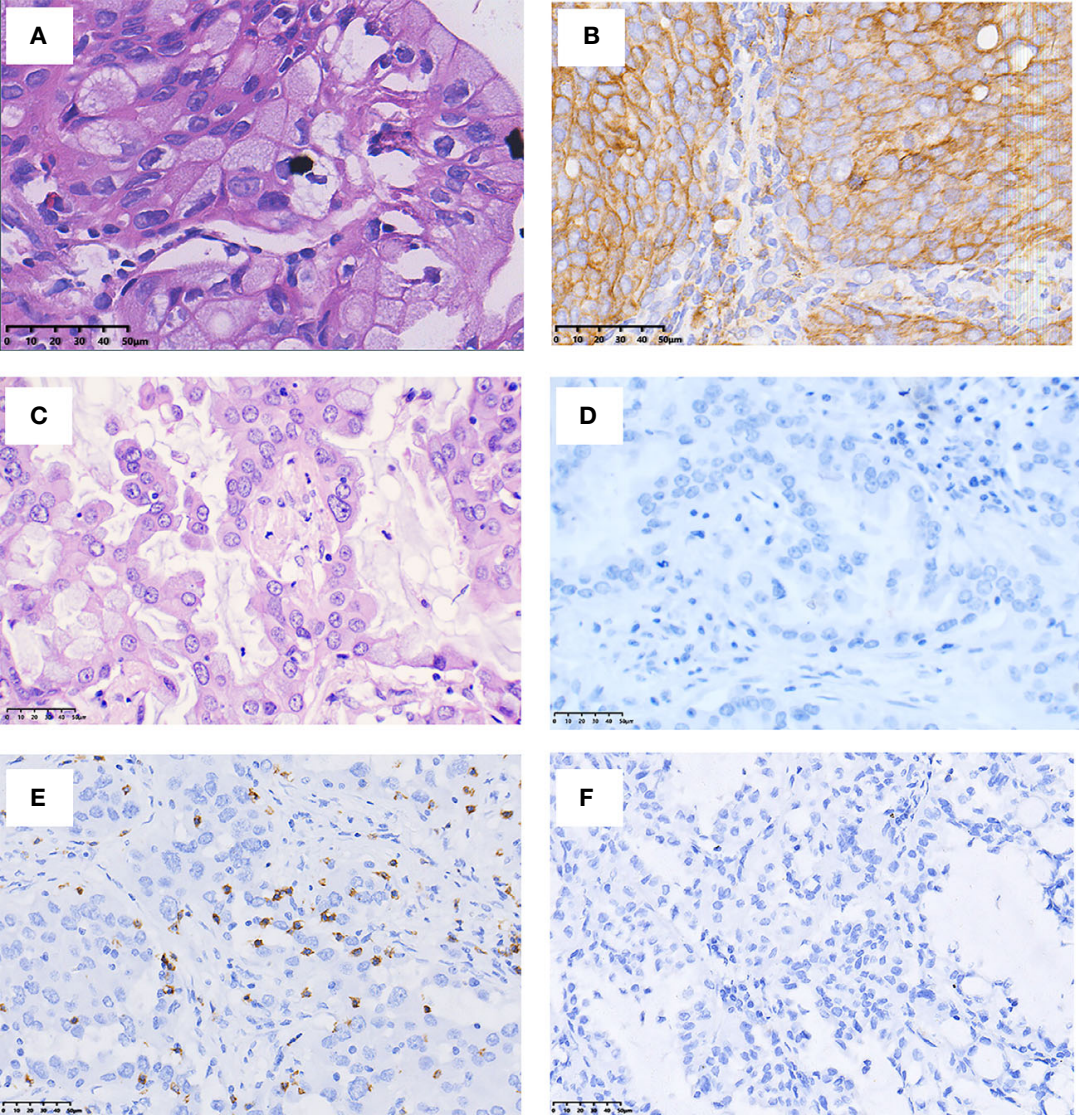
Representative images of PD-L1 and CD8 immuno-staining (×400 original magnification). H&E **(A)** and PD-L1 **(B)** staining of patients with PD-L1+; H&E **(C)** and PD-L1 **(D)** staining of patients with PD-L1−; **(E)** presence of CD8+ TILs; and **(F)** absence of CD8 + TILs.

**Figure 3:**
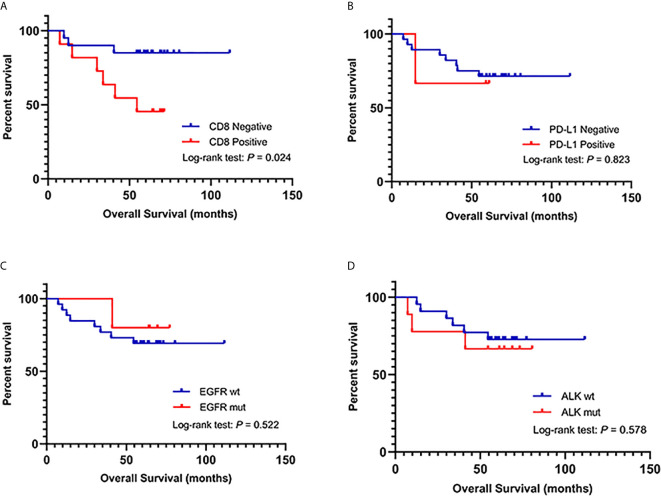
**(A)** Kaplan–Meier analysis of overall survival (OS) in IMA patients based on CD8+ TIL infiltration. **(B)** Kaplan–Meier analysis of OS in IMA patients based on PD-L1 expression. **(C)** Kaplan–Meier analysis of OS in IMA patients based on EGFR mutations. **(D)** Kaplan–Meier analysis of OS in IMA patients based on ALK mutations.

**Figure 2** presents the representative images for PD-L1 expression on the membrane of tumor cells and CD8+ TILs. As shown in **Table 1**, 9.7% (3/31) of the IMA patients had positive expression of PD-L1, and 35.5% (11/31) showed positive CD8 staining, which were markedly lower than that of non-IMA group [PD-L1: 48.1% (13/27); CD8: 81.5% (22/27)]. Differences between the groups were statistically significant (P < 0.001). Neither the PD-L1 expression nor CD8+ staining showed the association with other clinical factors (**Tables S1**, **S2**). The median OS was significantly shorter in patients with CD8+ staining than in those obscene (47.3 *vs* 60.2 months, P = 0.024, **Figure 3A**). As presented in **Table 2**, CD8 TIL status was correlated with poor OS (HR = 4.32, 95% CI: 1.08–17.34, P = 0.039) by the univariate analyses. Furthermore, after adjusting for clinicopathological factors, multivariate analysis suggested that CD8 was an independent prognostic factor for survival (HR = 5.60, 95% CI: 1.35–23.22, P = 0.017). Neither the PD-L1 expression nor the mutations of EGFR, ALK showed prognostic value (P > 0.05). Neither the PD-L1 expression nor the mutations of EGFR, ALK showed prognostic value (P > 0.05, **Figures 3B–D**)

**Table 2:** Univariate and multivariate Cox regression analyses of prognostic factors for survival in patients with IMA.

	Univariate analysis		Multivariate analysis	
	HR (95% CI)	*P*-value	HR (95% CI)	*P-*value
Sex				
Male	1.00	0.825	1.00	0.794
Female	1.16 (0.31–4.32)		0.81 (0.18–3.79)	
Age				
<65	1.00	0.444	1.00	0.290
≥65	0.44 (0.06–3.55)		0.31 (0.03–2.74)	
EGFR status				
Wild	1.00	0.586	1.00	0.620
Mutation	0.56 (0.70–4.49)		0.58 (0.07–4.90)	
ALK status				
Wild	1.00	0.658	1.00	0.646
Mutation	1.37 (0.34–5.48)		1.46 (0.29–7.30)	
PD-L1 expression				
+ (≥1%)	1.00	0.824	1.00	0.534
- (<1%)	0.79 (0.10–6.33)		0.49 (0.05–4.65)	
CD8 expression				
+ (≥10%)	1.00	0.039*	1.00	0.017*
- (<10%)	4.32 (1.08–17.34)		5.60 (1.35–23.22)	

HR, hazard ratio; CI, confidence interval.

*P-value < 0.05 in Cox proportional hazard model. In bold: P < 0.05.

### Response to Immunotherapy of IMA

**Figure 4:**
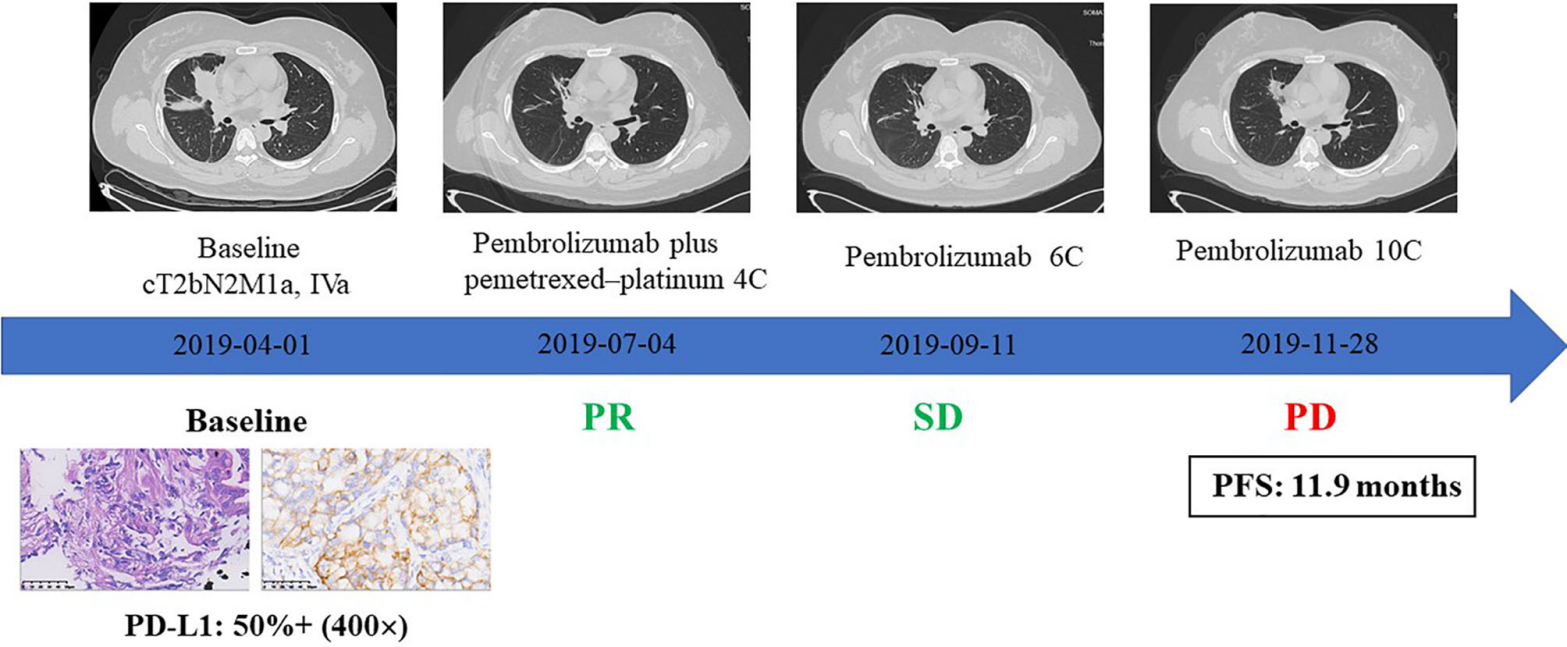
The clinical response to immune checkpoint inhibitors combined with chemotherapy in an IMA patient with PD-L1 expression and CD8+ TIL infiltration.

To evaluate the clinical efficacy of checkmate inhibitor based on TME status, we collected clinical data of a patient with IMA of the lung who was treated with PD-1 inhibitors. As shown in **Figure 4**, the patient was a 32-year-old young woman with lung ADC with pleural metastases (cT2bN2M1a, stage IVA). The IHC of her biopsy sample showed positive PD-L1 expression (50%) and strong CD8+ staining (40%). After four cycles of pembrolizumab plus pemetrexed–platinum regimen, the patient exhibited partial response (PR), with obviously shrunken pulmonary lesions (**Figure 4**). This patient then continued to receive pembrolizumab monotherapy and experienced a disease stabilization. Ultimately, after six cycles of pembrolizumab monotherapy, the patient developed disease progression due to a relapse with lung lesions; PFS was of 11.9 months.

## Discussion

As ICIs have dramatically changed anticancer strategies recently, there is a great need to better understand the immune axis and crosstalk with the TME in tumors. To our knowledge, this is the first study to evaluate the expression of PD-L1 and the infiltration of lymphocytes in patients with IMA. By reviewing the clinicopathological data of 31 IMA patients, we mainly investigate the immunophenotypic characteristics and their clinical relevance in IMA patients.

First, our study found a statistically significant decrease in PD-L1 expression and CD8+ TIL infiltration in IMA patients compared to non-IMA patients. Similar to our results, previous studies have also shown a low level of PD-L1 expression in IMA group (7, 17, 18). However, studies regarding TIL abundance in patients with IMA have not been carried out yet, which need to be verified by experiments with large sample sizes. As far as we know, interferon-*γ* (IFN-*γ*) can up-regulate PD-L1 expression through the JAK2–STAT1 and PI3K–AKT pathways in NSCLC (19, 20). Considering that CD8+ TILs could produce IFN-*γ* and induce PD-L1 expression (21), we speculate that the decrease of PD-L1 expression in IMA patients is associated with low levels of CD8+ TIL infiltration by inhibiting IFN-*γ* production. However, the mechanism underlying low CD8+ TIL infiltration in IMA needs further investigations.

Moreover, in the present study, 16.1% of IMA patients have EGFR mutations and 29.0% with ALK rearrangement, which was consistent with previous studies indicating that IMA patients have a lower frequency of EGFR mutations and a higher frequency of ALK rearrangements than non-IMA patients (8, 9, 12). Our former next-generation sequencing (NGS)-based study using a panel of 425 genes has identified that KRAS mutations were the most frequent oncogenic driver mutations in IMAs (23.1% in pure-IMA group and 4.0% in mixed-IMA group) (22). However, owing to the rarity or absence of targetable mutations, there are few studies on the target therapy of IMA (23). Several studies have suggested that KRAS mutations, ERBB2 mutations, and ALK rearrangements could be targeted for therapeutic intervention; however, its efficacy for IMA patients has not be verified in clinical practice yet (24, 25). Since NRG1 fusions are in a high proportion of lung IMA, multiple studies have demonstrated that afatinib, an irreversible ErbB family inhibitor, is effective in NSCLC patients with NRG1 fusions, which maybe a potential therapeutic strategy to IMA patients (26–28). Hence there is an urgent need to determine the molecular mechanisms driving IMA and identify novel target therapy.

Second, we observed that the CD8-negative group exhibited longer overall survival (OS) than CD8+ TIL group, and CD8 was an independent prognostic factor for IMA patients’ survival. Contrary to our results, CD8+ T lymphocytes are thought to be the dominant cytotoxic immune cells that are able to eliminate tumor cells (29). Recently, elevated levels of cytotoxic CD8+ T cells in the TME have been linked with positive anti-tumor effects in various cancers, indicating a good prognosis in patients with elevated cytotoxic CD8+ TILs (30–32). Researchers have found that CD8+ TILs at different tumor sites have diverse clinical attributes and a number of factors in the TME, such as the activation of inhibitory checkpoint pathways, abnormal tumor angiogenesis, and chemokine secretions, can exactly suppress the function of CD8+ TILs (33). Thus, we speculate that IMA patients have a special TME that affect CD8+ TILs.

Our results showed that IMA patients have low PD-L1 expression and less CD8+ infiltration, which indicated poorer response rates to checkmate inhibitors (34, 35). Therefore, we considered that patients with IMA cannot benefit from ICI monotherapy; surgery and platinum-based conventional chemotherapy are still the main therapeutic modalities. In our study, one female patient with strong PD-L1 expression (50%) and abundant CD8+ T cell infiltration (40%) experienced PR to ICIs combined with chemotherapy. We speculate that the combination of immunotherapy and chemotherapy may be a new therapeutic direction for advanced IMA patients. In addition, the favorable clinical efficacy of ICIs is also associated with positive PD-L1 expression and CD8+ T cell infiltration in the tumor tissue, as sufficient CD8+ TILs in the TME is the foundation of anti-tumor effect activated by ICIs (36). However, the potential of PD-L1 and CD8 as predictive biomarkers to immunotherapy in IMA patients remains to be further investigated.

Our study has several limitations. First, the number of patients enrolled in this study was relatively small due to the rarity of IMA. Second, the IMA group was not further divided into pure-IMA and mixed-IMA subgroups, leading to the possible heterogeneity of the IMA group which may influence the expression of PD-L1 and CD8+ TILs. Third, some patients were diagnosed at an early stage, and the median OS in the IMA group was unavailable. Fourth, because of insufficient samples, ALK rearrangement was not reconfirmed by fluorescence *in situ* hybridization (FISH) assay. Finally, as the samples used in study were obtained years ago, the PD-L1 expression may be underestimated because the expression of PD-L1 might be dynamic with time going.

In conclusion, we demonstrated that IMA patients might have lower levels of PD-L1 expression and CD8+ TIL infiltration than non-IMA patients. We also observed a lower frequency of EGFR mutations and a higher frequency of ALK rearrangements and KRAS mutations in IMA patients. Moreover, patients with CD8+ TIL infiltration had shorter OS and worse prognosis, and CD8 was an independent prognostic factor of IMA patients’ survival. Based on the characteristics of gene and immune microenvironment, target therapy and immunotherapy in IMA patients are limited, which needs further investigation.

## Data Availability Statement

The datasets presented in this study can be found in online repositories. The names of the repository/repositories and accession number(s) can be found in the article/**Supplementary Material**.

## Ethics Statement

The studies involving human participants were reviewed and approved the Ethics Committee of Zhejiang Cancer Hospital (IRB−2021−14). Written informed consent was obtained from all recruited participants.

## Author Contributions

Conceptualization: YF. Methodology: XX and DW. Investigation: NL. Bioinformatics analysis: XX and NL. Writing, original draft: NL. Writing, review, and editing: WC. Funding acquisition: XX. Supervision: YF. All authors contributed to the article and approved the submitted version.

## Funding

This work was supported by grants from the National Natural Science Foundation of China [81802995 and 81672315], Zhejiang Province Public Welfare Funds [LGF19H280004].

## Conflict of Interest

The authors declare that the research was conducted in the absence of any commercial or financial relationships that could be construed as a potential conflict of interest.
