# Supplementary figures and images for: Clinical Relevance of PD-L1 Expression and CD8+ T Cells’ Infiltration in Patients With Lung Invasive Mucinous Adenocarcinoma
Source: Front Oncol. 2021 Jun 24;11:683432. doi: 10.3389/fonc.2021.683432 (PMC8264667; doi:10.3389/fonc.2021.683432)

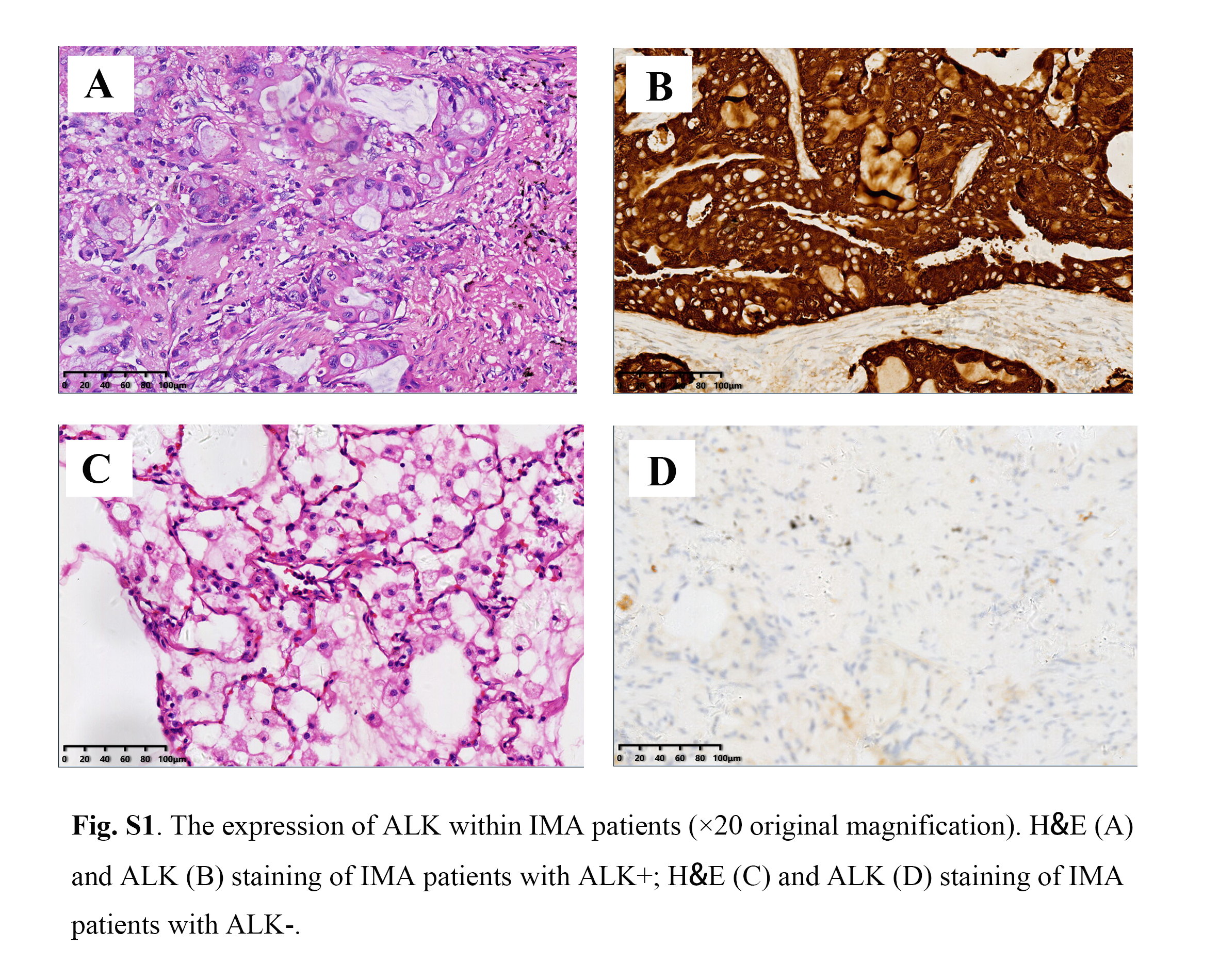

Supplement: Supplementary file 1 [file Image_1.tif]

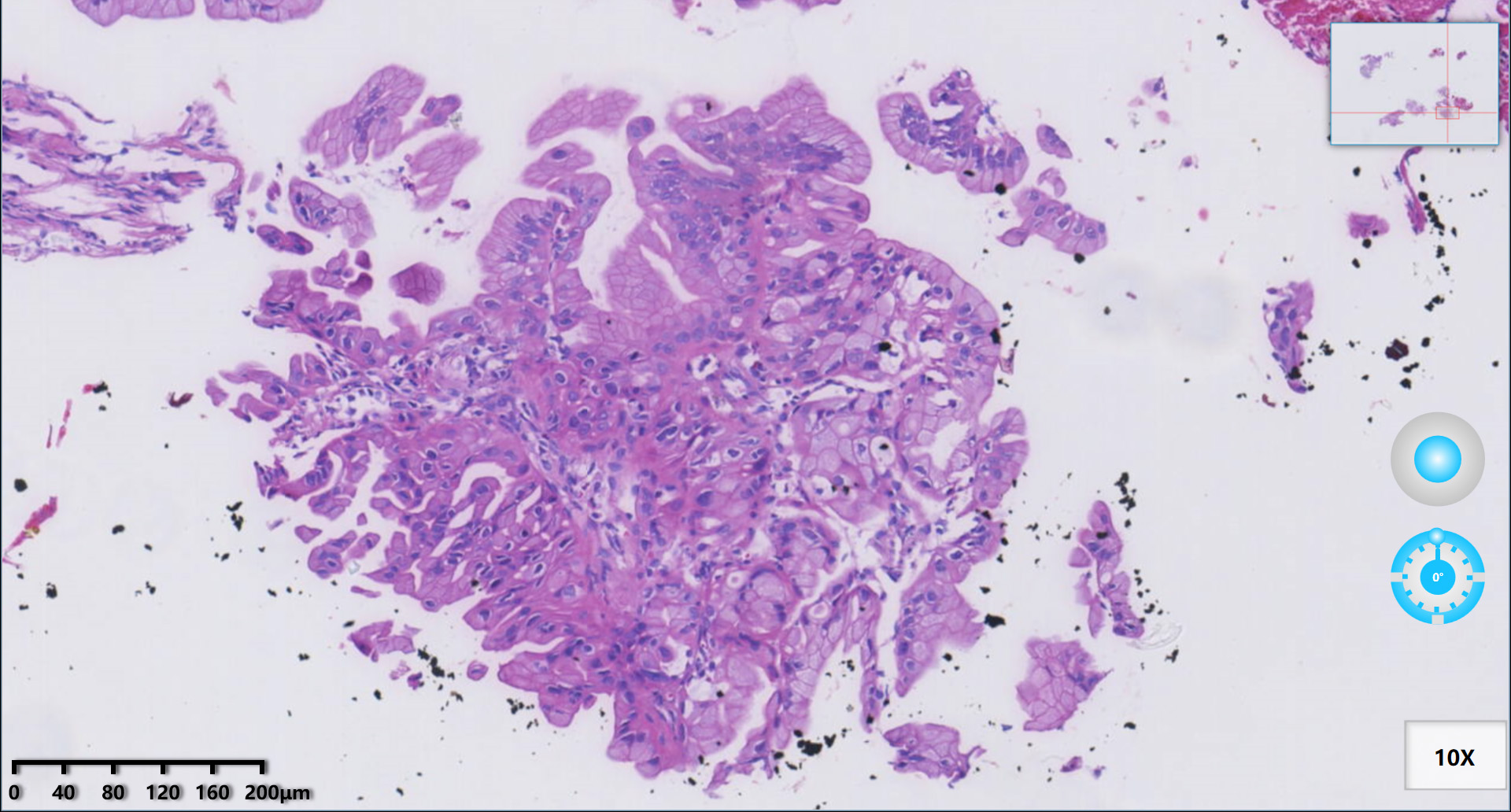

Supplement: Supplementary file 2 [file Image_2.jpeg]
