# Supplementary material for: Clinical Relevance of PD-L1 Expression and CD8+ T Cells’ Infiltration in Patients With Lung Invasive Mucinous Adenocarcinoma
Source: Front Oncol. 2021 Jun 24;11:683432. doi: 10.3389/fonc.2021.683432 (PMC8264667; doi:10.3389/fonc.2021.683432)
Supplement: Supplementary file 3 [file Table_1.docx]

Table S1. The association of CD8+ TILs infiltration with clinical factors in IMA patients.

|  | All case  (n = 31) | CD8+  (n = 11) | CD8-  (n = 20) | *P*-value |
| --- | --- | --- | --- | --- |
| Sex |  |  |  |  |
| Male | 16 (51.6%) | 7 (43.8%) | 9 (56.3%) | 0.32 |
| Female | 15 (48.4%) | 4 (26.7%) | 11 (73.3%) |  |
| Age |  |  |  |  |
| <65 | 25 (80.6%) | 8 (32.0%) | 17 (68.0%) | 0.408 |
| ≥65 | 6 (19.4%) | 3 (50.0%) | 3 (50.0%) |  |
| Smoking status |  |  |  |  |
| Never | 19 (61.3%) | 6 (31.6%) | 13 (68.4%) | 0.567 |
| Ever/current | 12 (38.7%) | 5 (41.7%) | 7 (58.3%) |  |
| Clinical stage |  |  |  |  |
| I-III | 27 (87.1%) | 11 (40.7%) | 16 (59.3%) | 0.112 |
| IV | 4 (12.9%) | 0 (0.0%) | 4 (100.0%) |  |
| EGFR status |  |  |  |  |
| Wild | 26 (83.9%) | 9 (34.6%) | 17 (65.4%) | 0.818 |
| Mutation | 5 (16.1%) | 2 (40.0%) | 3 (60.0%) |  |
| ALK status |  |  |  |  |
| Wild | 22 (15.5%) | 8 (36.4%) | 14 (63.6%) | 0.873 |
| Mutation | 9 (82.8%) | 3 (33.3%) | 6 (66.7%) |  |
| PD-L1 expression |  |  |  |  |
| - (<1%) | 28 (90.3%) | 10 (35.7%) | 18 (64.3%) | 0.935 |
| + (≥1%) | 3 (9.7%) | 1 (35.5%) | 2 (64.5%) |  |

P-value in Chi-square test
