# Supplementary material for: Clinical Relevance of PD-L1 Expression and CD8+ T Cells’ Infiltration in Patients With Lung Invasive Mucinous Adenocarcinoma
Source: Front Oncol. 2021 Jun 24;11:683432. doi: 10.3389/fonc.2021.683432 (PMC8264667; doi:10.3389/fonc.2021.683432)
Supplement: Supplementary file 4 [file Table_2.docx]

Table S2. The association of PD-L1 expression with clinical factors in IMA patients.

|  | All case (n = 31) | PD-L1+ (n = 3) | PD-L1- (n = 28) | *P*-value |
| --- | --- | --- | --- | --- |
| Sex |  |  |  |  |
| Male | 16 (51.6%) | 2 (12.5%) | 14 (87.5%) | 0.583 |
| Female | 15 (48.4%) | 1 (6.7%) | 14 (93.3%) |  |
| Age |  |  |  |  |
| <65 | 25 (80.6%) | 2 (8.0%) | 23 (92.0%) | 0.519 |
| ≥65 | 6 (19.4%) | 1 (16.7%) | 5 (83.3%) |  |
| Smoking status |  |  |  |  |
| Never | 19 (61.3%) | 1 (5.3%) | 18 (94.7%) | 0.296 |
| Ever/current | 12 (38.7%) | 2 (16.7%) | 10 (83.3%) |  |
| Clinical stage |  |  |  |  |
| I-III | 27 (87.1%) | 3 (11.1%) | 24 (88.9%) | 0.483 |
| IV | 4 (12.9%) | 0 (0.0%) | 4 (100.0%) |  |
| EGFR status |  |  |  |  |
| Wild | 26 (83.9%) | 3 (11.5%) | 23 (88.5%) | 0.424 |
| Mutation | 5 (16.1%) | 0 (0.0%) | 5 (100.0%) |  |
| ALK status |  |  |  |  |
| Wild | 22 (15.5%) | 3 (13.6%) | 19 (86.4%) | 0.244 |
| Mutation | 9 (82.8%) | 0 (0.0%) | 9 (100.0%) |  |
| CD8 expression |  |  |  |  |
| - (<1%) | 20 (64.5%) | 2 (10.0%) | 18 (90.0%) | 0.935 |
| +(≥1%) | 11 (35.5%) | 1 (9.1%) | 10 (90.3%) |  |

P-value in Chi-square test
